# Wood properties of *Carapa guianensis* from floodplain and upland forests in Eastern Amazonia, Brazil

**DOI:** 10.1038/s41598-019-46943-w

**Published:** 2019-07-23

**Authors:** Anderson Vasconcelos Firmino, Graziela Baptista Vidaurre, José Tarcísio da Silva Oliveira, Marcelino Guedes, Maria Naruna Felix de Almeida, João Gabriel Missia da Silva, João Vicente de Figueiredo Latorraca, José Cola Zanuncio

**Affiliations:** 1 Departamento de Ciências Florestais e da Madeira, Universidade Federal do Espírito Santo, Jerônimo Monteiro, 29550-000 Brazil; 2 Centro de Pesquisa Agroflorestal do Amapá, Empresa Brasileira de Pesquisa Agropecuária, Macapá, 68903419 Brazil; 3 Departamento de Produtos Florestais, Universidade Federal do Rio de Janeiro, Seropédica, 23851-970 Brazil; 4 Departamento de Entomologia/BIOAGRO, Universidade Federal de Viçosa, Viçosa, 36570-900 Brazil

**Keywords:** Ecophysiology, Forest ecology

## Abstract

The variable environmental conditions of the Amazon forest can affect the wood properties of the tree species distributed across its diverse phytophysiognomies. *Carapa guianensis* (Andiroba) occurs in upland and floodplain forests, and the wood and oil of its seeds have multiple uses. The aim of this study was to evaluate the wood properties of *C. guianensis* trees in upland and estuarine floodplain forests of the Amazon River. Eight trees were selected, with four being from the upland and four from the floodplain forests. The fiber length, fiber wall thickness, vessel diameter and frequency, microfibril angle, specific gravity and wood shrinkage were evaluated. The juvenile and mature wood zones were determined according to these variables. The fiber length, fiber wall thickness and specific gravity increased, and microfibril angle decreased, in the pith to bark direction. Only the fiber length variable was efficient for delimiting juvenile, transition and mature wood. The fiber length, wall thickness and specific gravity of wood were higher in upland forest trees. However, the environment did not alter the beginning of the formation and proportion of *C. guianensis* mature wood. This information is important for the log fit in cutting diagrams, aiming toward improving the production, classification and processing of pieces with specific quality indexes in order to direct them to appropriate wood uses.

## Introduction

The Amazon is the largest biome in Brazil, occupying almost half of the territory, and the largest world biodiversity reserve. This biome has one third of the planet’s tropical forests (4.2 million km^2^) and numerous phytophysiognomies^1^, including floodplain and upland forests. Floodplain forests are characterized by low and flooded terrains and with a seasonal and/or daily variation of river water level^2^, while the upland forests are found in higher areas without floods influence^3^. Soil fertility is higher in floodplain areas favoring tree growth^4^, while precipitation seasonality and lower soil fertility are characteristic of upland areas^5^.

*Carapa guianensis* (Meliaceae), popularly known as Andiroba, has high economic value and this tree is found in upland and floodplain forests. The tree has multiples uses and its wood has high value for solid products including furniture manufacturing, construction, veneers and plywood; and the oil of the seeds, besides medicinal importance^6–8^.

The use and sustainable management of *C. guianensis* wood depend on the knowledge of wood properties, such as the percentage of mature wood as one of the main indicators of wood quality. The definition of juvenile and mature wood help decisions on minimum harvesting age and processing methodologies optimizing timber production and reducing environmental impacts. Mature wood, developed in the mature stage of the vascular cambium, has more uniform properties and greater mechanical resistance, with better characteristics for sawmill and structural purposes^9–11^. Wood properties such as fiber length and wall thickness^10,12^, microfibril angle^13^, width of the growth ring^14^, specific gravity^15^ and shear modulus^16^ are used to define the transition between juvenile and mature wood.

Environmental^17^, edaphic^18,19^ and hydric availability^20^ conditions affect wood quality. Almost twice as many *C. guianensis* trees were found in occasionally flooded forests compared to dry forests^7^, making it necessary to study the properties of *C. guianensis* wood in both environments. However, the wood harvesting s is easier on upland than in flooded areas. The aims of this study were to evaluate the anatomical and physical wood properties, to identify changes between juvenile, transition and mature *C. guianensis* wood from trees in upland and flooded areas in the Amazonian region of Brazil, help management plans for this plant and its wood processing.

## Results

### Variation of the anatomical characteristics and wood specific gravity along the pith-to-bark profile

**Figure 1:**
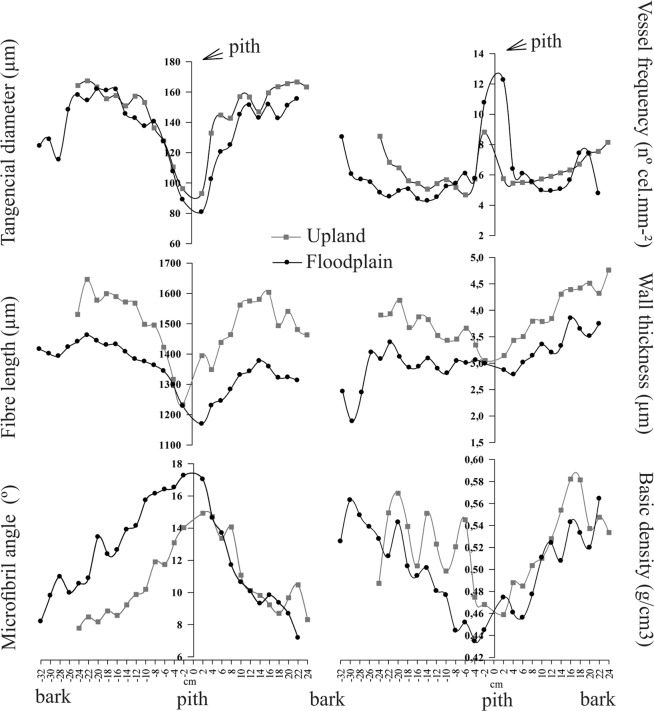
Variation in the bark-to-bark direction of the anatomical elements and wood specific gravity of *Carapa guianensis* trees in upland and floodplain forests in Eastern Amazonia, Brazil. The lines are means of four samples for each environment.

The proprieties, vessel diameter, fiber length, wall thickness and wood specific gravity had a common wood pattern variation, meaning they increased along the pith-to-bark profile when the microfibril angle decreased. The vessel frequency was higher near the pith, decreasing in the intermediate position and increasing near the bark. The fiber length, wall thickness and specific gravity values had similar trend in both forests, but with lower values in the wood from floodplain trees, while the microfibril angle was smaller in upland forest trees (Fig. 1).

### Wood properties in trees from upland and floodplain forests

The αt/αr ratio of *C. guianensis* wood was similar between environments, while most properties showing higher values in upland forests. The specific gravity, fiber wall thicker, pores frequency and diameter were higher, and the microfibril angle lower in upland forest trees (Table 1). The coefficient of variation for the wall thickness, vessel diameter, microfibril angle, specific gravity, αt/αr and tangential/radial shrinkage were higher in the wood from floodplain trees. Fiber length was the most homogeneous property between trees of both environments and with a lower variation coefficient.

**Table 1:** Wood anatomical characteristics and wood physical properties from *Carapa guianensis* trees of upland and floodplain forests in Eastern Amazonia, Brazil.

Variables	Upland forest		Floodplain forest	
	Mean	CV (%)	Mean	CV (%)
Fiber length (μm)	1505.27**	7.0	1356.40	5.3
Fiber wall thickness (μm)	3.73**	8.3	3.12	16.0
Vessel tangential diameter (μm)	144.82**	9.7	132.21	13.9
Vessel frequency (n° cel mm^−^²)	6.39**	12.3	5.87	9.7
Microfibril angle (°)	11.31	11.31	12.93**	20.0
Specific gravity (g cm^−3^)	0.52**	11.0	0.49	12.5
Tangential shrinkage (%)	8.23**	8.23	7.16	16.4
Radial shrinkage (%)	4.97**	4.97	4.27	27.1
αt/αr ratio	1.70	1.70	1.45	27.0

**mean values of parameters differ between *Carapa guianensis* wood of upland and floodplain forests by Student’s t-test at 1%.

### Delimitation of juvenile, transition and mature wood

The fiber length was efficient to delimiting juvenile, transition and mature *C. guianensis* wood in both environments. The non-significant linear regression and low determination coefficients for the diametric variation of specific gravity, fiber wall thickness, vessel diameter and frequency, microfibril angle and shrinkage did not allow using these parameters to delimit juvenile from mature wood.

**Figure 2:**
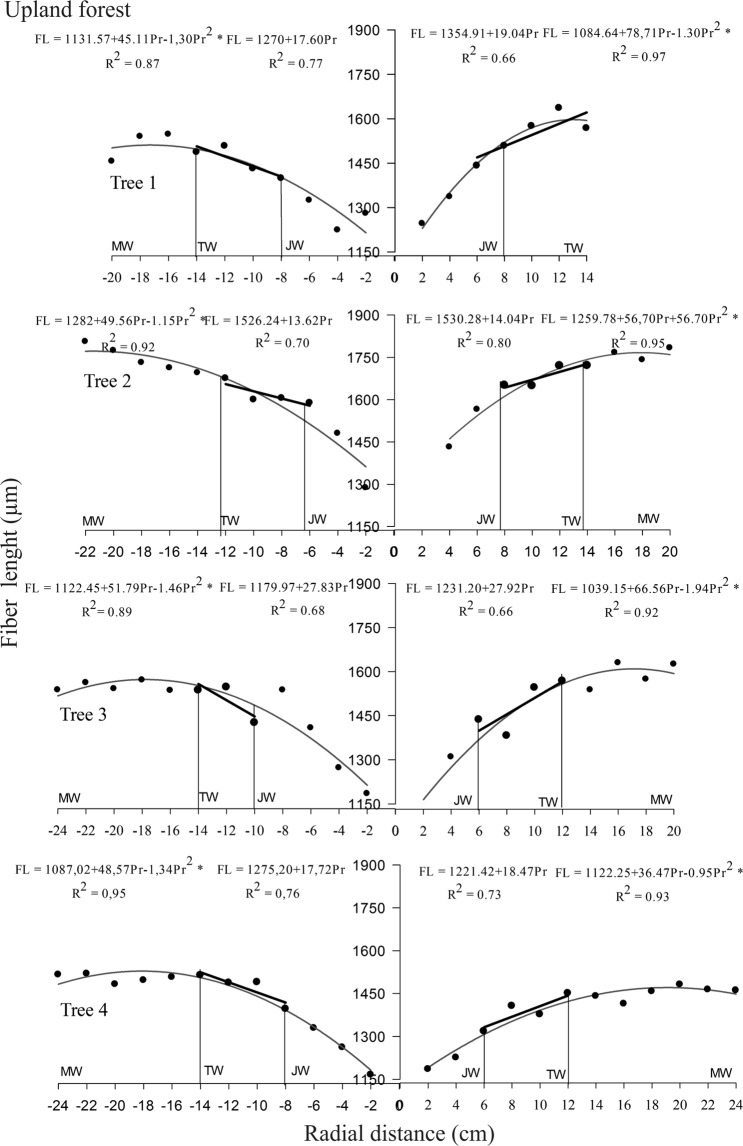
Delimitation between juvenile (JW), transition (TW) and mature (MW) wood in *Carapa guianensis* trees from upland forest, Eastern Amazonia, Brazil. (*) Significant regression at 5% probability.

**Figure 3:**
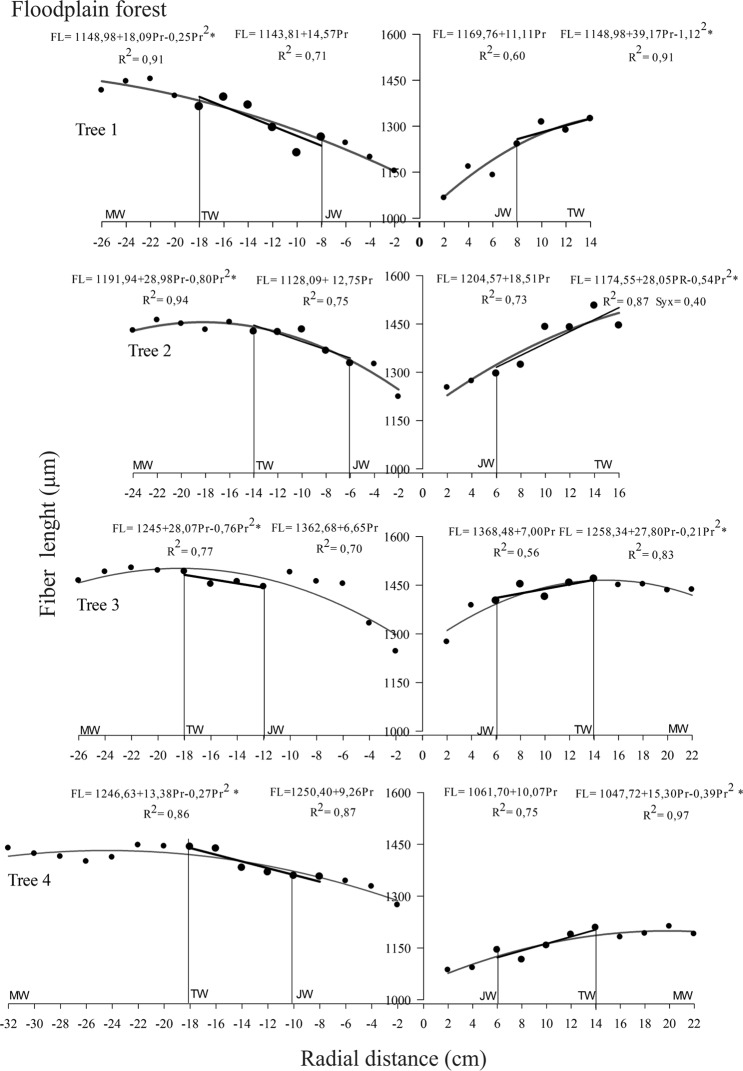
Delimitation between juvenile (JW), transition (TW) and mature (MW) wood in *Carapa guianensis* trees from floodplain forest, Eastern Amazonia, Brazil. (*) Significant regression at 5% probability.

Fiber length increased linearly from the pith up to approximately 6 to 10 cm in upland trees and from 6 to 12 cm in those in flooded areas, defining the juvenile wood zone. Two zones were distinguished (Figs 2 and 3) after this region; the first, characterizing the transition zone between the juvenile and mature wood with a linear but less marked increase in fiber length of between 6 and 14 cm and 8 to 18 cm in the pith to bark profile for upland and floodplain trees, respectively. The mature wood zone was the second and more constant and stable growth characteristic. The proportion of each wood type was similar in both environments, but the mature wood quantity in both forests was lower than that of juvenile and transition wood combined (Table 2).

**Table 2:** Proportion of juvenile, transition and mature wood in *Carapa guianensis* trees from upland (UF) and floodplain (FF) forests in Eastern Amazonia, Brazil.

Tree	Proportion					
	Juvenile wood		Transition wood		Mature wood	
	UF	FF	UF	FF	UF	FF
1	47	40	35	40	18	20
2	33	30	29	45	38	25
3	36	38	23	29	41	33
4	29	30	25	30	46	41
Mean	36 ^ns^ (21.3)	34 (15.2)	28^ns^ (18.9)	36 (21.3)	36^ns^(34.3)	30 (30.9)

ns: not significant. Mean of proportion of juvenile, transition and mature wood of *Carapa guianensis* do not differ between upland and floodplain forests by Student’s t-test at 1%. Values in parentheses represent the coefficient of variation.

### Properties of juvenile, transition and mature wood

Vessel frequency, tangential and radial shrinkage, and consequently the αt/αr ratio, were similar between juvenile, transition and mature wood. The specific gravity, fiber length, wall thickness and vessel diameter of juvenile wood were lower for wood of both environments, similar to the natural tendency of these wood properties with lower values for juvenile wood than to mature wood (Table 3). The wood of upland trees had fiber length and wall thickness with higher increase from juvenile to mature wood, and the microfibril angle presented a greater reduction in upland wood.

**Table 3:** Fiber length (F.L. μm), fiber wall thickness (F.W.T. μm), vessel diameter (V.T.D. μm), vessel frequency (V.F.n°. mm^−2^), Microfibril angle (M.F.A.), specific gravity (S.G. g cm^−3^), tangential shrinkage (T.S.%), radial shrinkage (R.S.%), αt/αr ratio (αt/αr) of juvenile wood (JW), transition wood (TW) and mature wood (MW) wood and percentage of increase or decrease of these properties between the juvenile and mature wood of *Carapa guianensis* trees of upland forest and floodplain forest, Eastern Amazonia, Brazil.

Var.	Upland forest				Floodplain forest			
	JW	TW.	MW	%	JW	TW	MW	%
F.L.	1357.73c	1542.50b	1613.40a	16	1267.89b	1374.24a	1370.63a	7
F.W.T.	3.46c	3.72b	4.21a	18	2.97b	3.14b	3.44a	14
V.T.D.	120.22c	151.12b	160.79a	25	108.77b	146.10a	150.64a	28
V.F.	6.20a	5.28b	6.02a	−3	7.58a	4.91c	6.03b	−20
M.F.A.	13.32a	10.45b	8.51c	−36	15.87a	12.46b	10.72b	−32
S.G.	0.50b	0.52b	0.55a	9	0.46b	0.49b	0.53a	13
T.S.	8.00a	8.80a	8.00a	0	7.06a	7.33a	7.09a	0
R.S.	5.28a	4.75a	5.22a	−1	4.46a	4.18a	4.00a	−10
αt/αr	1.54b	1.94a	1.65ab	7	1.91a	1.92a	1.90a	−1

Variables (Var.), %: increase or decrease the values of juvenile wood properties for the mature wood. Average followed by the same letter in the line do not differ at the 5% significance by the Tukey test.

## Discussion

Internal variations of upland trees wood demonstrate that properties such as specific gravity^21^, fiber length and wall thickness^22^ increased from pith-to-bark profile, while the microfibril angle^22,23^ decreased. Differences in values between floodplain and upland forests show that soil and environmental conditions decreased the specific gravity, fiber length values while increased those of wall thickness of floodplain plants. Besides, the shape of the fiber length curve from pith-to-bark was also different among the two habitats. Differences in cellular dimensions of anatomical elements are associated to wood maturation^24^, and are due to a combination of anatomical, physical and chemical factors, mainly influenced by tree age, genotype and environmental growth conditions^25–27^. It is difficult isolate these factors in natural growth forests. *C. guianensis* does not present distinct growth rings^28^, what difficult the determination of its trees’ age by dendrochronology. Tree age influences the wood properties^29^, and thus this factor has potential to confound comparisons between floodplain and upland trees. The sampling by diameter class aimed to select individuals with similar diametric development to reduce the lack of knowing the tree age helping to maintain the sample standard.

The lower wood specific gravity of *C. guianensis* wood from floodplain areas agrees with that reported for 58 tree species in the Sustainable Development Reserve of Mamirauá in the Central Amazon, Brazil^20^. The higher wood specific gravity of *C. guianensis* wood from upland forests represents a higher mass per volume, number of hydrophilic sites and water adsorption capacity. This results in higher tangential and radial shrinkage^30^, similar to the direct relationship between wood specific gravity and shrinkage of *Eucalyptus urophylla* and *Corymbia citriodora*^31,32^.

The lower fiber length and wall thickness values of *C. guianensis* trees in flooded environments may be associated to reduced metabolism and plant development, with structural and physiological adaptations to the lower oxygen levels for the roots^33^. Moreover, as a perennial species, the sap flow of *C. guianensis* is lower than that of deciduous species. This behavior compensates for seasons with lower water availability through stomatal control and water storage in the xylem^34^. This may be associated with a larger dimension of the anatomical elements in wood of the upland area, allowing the trees to maintain their development during drought periods in areas without flooded.

The lower wood property of *C. guianensis* wood from trees of the flooded environments are similar to those reported for *Croton urucurana*, with lower fiber length and wall thickness^35^, *Calophyllum Brasiliense*, with lower vessel frequency^36^ and *Chorisia speciosa*, with a smaller vessel diameter with flooding for 45 days^37^.

Delimiting juvenile and mature *C. guianensis* wood by fiber length confirms the importance of this parameter as the main characteristic to distinguish them, and is also used for other tropical hardwoods^12,22^. The highest annual increase rate in fiber length in the early years of tree growth is characteristic of juvenile wood formation^10,12,38^. Mature wood has more stable property values than juvenile ones^39–41^, reflecting in better quality pieces and added value. Aiming to delimit these regions more precisely, the limit between juvenile and mature wood was identified in individual trees. Adopting a specific fiber length value for all trees is risky, because it can attribute the same quality for juvenile and adult wood, only being based on achieving predetermined values.

Proportion of juvenile wood in *C. guianensis* trees from upland forests was similar to that of hardwoods from homogeneous plantations in Brazil such as *Hevea brasiliensis*^10^ and *Eucalyptus grandis*^12^, occupying 30 to 40% of the diameter. This similarity reinforces the *C. guianensis* tolerance to flooding and its adaptability to different environmental conditions with reduced physiological alterations and low impact on the maturation of the cambium and mature wood formation. The similarity in chlorophyll and total carotenoid levels in *C. guianensis* plants, with availability of water or not, indicates that drought does not degrade leaf pigments in this plant^42^. Tolerant species, such as *C. guianensis*, maintain their normal roots or produce side and adventitious ones in areas with flooding^43^.

The greater growth of trees in floodplain forest with mature wood proportions similar to those of upland is expected a greater exploitation of the first forests in spite of the harvesting being easier on upland areas. However, the lower wood density in the floodplain forest suggests a longer tree cutting cycle, and consequently a lower harvesting density due to selecting trees with wood quality. This makes necessary to reinforce the importance of harvesting in both environments without exceeding the maximum productivity/ha, while conserving important trees to disperse seeds to maintain the genetic variability.

Small fibers with thin walls, smaller vessel diameter, lower specific gravity, larger microfibril angle and lower volumetric stability of *C. guianensis* juvenile wood in upland and floodplain forests are due to the initial cambial cell size increase according to the age of the secondary meristem, becoming more stable with wood maturity^24^.

The lower absolute values of specific gravity and fiber length and wall thickness in *C. guianensis* wood from floodplain trees can be explained by the formation of higher mechanical support (higher specific gravity and longer thicker fibers) in upland plants. Native trees of tropical forests invest differently in mechanical support and sap transport according to environmental conditions. Fractions of fiber length, lumen diameter and wall thickness (support related) vary between environments, with dry and nutrient poor habitats, selecting species with high mechanical support^44^. In addition, the higher altitude of upland forest makes trees more by winds. This increases the need for mechanical support, but no differences were observed in shape and size of the tree crown between the environments^45^.

The increase in the specific gravity from juvenile to mature wood in *C. guianensis* trees in both forests is important because specific gravity is the most commonly property used to assess wood quality because it is easy to measure and its related with drying^32^, dimensional stability^46^ and mechanical resistance^47^. Increasing wood specific gravity makes the mature wood more attractive for structural purposes. This increase was lower than that reported for *E. grandis* wood at 30-years-old^48^, and it is associated with the slower growth rate of the *C. guianensis* plants^49^, reducing the differences between the radial wood and making the wood more homogeneous.

The proportion of mature wood that can be obtained in a *C. guianensis* log is important for the timber sector. The diameter of 50 cm of the upland and floodplain trees is important for wood processing to optimize operations (fit of log cutting models) and planning the production (classification and separation of pieces). The uniformity of the pieces is important to use the wood as a solid product and the delimitation of juvenile and mature wood allows us to separate pieces of sawn wood according to their properties, thereby avoiding inappropriate use^50^.

The environmental agencies can use these proportions as theoretical scientific basis to determining the ideal minimum cutting diameter to increase the production quality. However, the extraction of high quality or selective wood has negative effects on the populations harvested. Minimum cut diameter (MCD), most often 50 cm DBH, are too low to preserve populations, and harvesting intensity and ecological processes should be considered to maintain forest structure and composition, and specie genetic quality and variability^51^. The MCD should be better studied^52^, and this research encourages allows to evaluate the technical and economic viability of MCD increase for this species in relation to the proportional increase of mature wood in log diameter and the ecological recovery period of the logging area.

## Conclusion

Wood variability (bark to bark direction) of *C. guianensis* in upland and floodplain forests was similar. The fiber length was the most adequate parameter to delimit the juvenile, transition and mature wood of this tree, because it presented greater differences and smaller coefficient of variation. Specific gravity, fiber length and radial and tangential shrinkage were higher in *C. guianensis* wood from upland forests.

Wood property values were lower in *C. guianensis* trees of the floodplain forest, but the formation age and proportion of mature wood were similar between environments. The proportion of mature wood in the stem and in the diameter bands studied in both environments was lower than that of juvenile and transition wood accounted for together.

## Material and Methods

### Tree selection and sampling

*C. guianensis* trees were harvested with authorization from the Chico Mendes Institute for Biodiversity Conservation (ICMBio- SISBIO 47586 -1) in the Cajari River Extractivist Reserve, in southern Amapá state, Brazil (0°15′S and 52°25′W, and 1°5′S and 51°31′W). The reserve area is 501,771 ha and includes the municipalities of Laranjal do Jarí, Mazagão and Vitória do Jari.

Eight trees with a minimum diameter at breast height (DBH) of 50 cm, according to the Brazilian legislation for forest management in the Amazon, were harvested, with four trees from the upland forest (medium DBH with bark 50.2 cm) and four from the floodplain forest (medium DBH with bark 55.0 cm). The diameter class of 45–55 cm were used as criteria for selecting the trees in both environments, as well as observations of the health and straightness of the stem, the height of the adventitious roots, the vitality of the crown and the low slope of the ground. The trees with presence of nests of birds or habitat of other animals and in seed production were disregarded.

**Figure 4:**
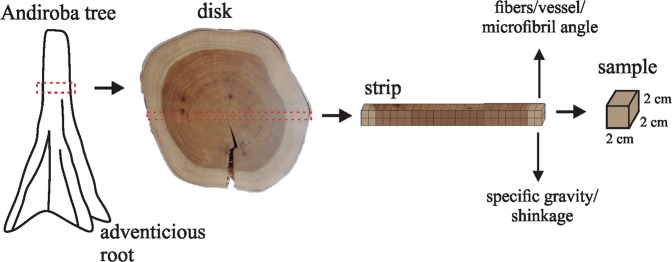
Disk and sample removal scheme for evaluation of the properties of *Carapa guianensis* wood from upland and floodplain forests.

A disk from each tree was removed above the region that finalized the adventitious roots, a common characteristic of this species. A cross-section in the bark to bark direction was marked, sectioned and subdivided into a 2 × 2 × 2 cm sample (radial, tangential and longitudinal directions) for evaluating the *C. guianensis* wood properties (Fig. 4). The rays with the greatest displacement of the pith, which possibly indicate the formation of reaction wood, were disregarded during the disk sampling.

### Morphology of fibers and vessels, microfibril angle and physical wood properties

Fiber length and wall thickness, vessel diameter and frequency were measured following the recommendations of *the International Association of Wood Anatomists* – IAWA^53^ using *AxioVision* 4. 9.1 software.

The microfibril angle was measured in 6 μm-thick, longitudinal tangential histological sections, using a sliding microtome. The fine wood sections were placed in a glass recipient covered with 1:1 acetic acid solution and hydrogen peroxide and stored in an oven at 60 °C for 12 hours for complete individualization of the fibers. The slices were washed in running water until the solution was completely removed and subsequently stored in distilled water.

The microfibril angle was measured by polarized light microscopy^54^ in a microscope equipped with a turntable and graduated from 0 to 360°. Twenty measurements were made for each position in the bark to bark direction under a 50 x magnification.

Specific gravity was determined by the ratio of dry mass to saturated volume in defect-free samples by immersion method in water according to the procedures of ASTM D2395^55^. The radial (αr) and tangential (αt) shrinkage of the wood were measured according to ASTM D143 in the same specific gravity samples^56^. The ratio between tangential and radial contraction (αt/αr) was also determined.

### Statistical analysis

The mean values of the properties between environments were compared with Student’s t-test.

The transition region between juvenile and mature wood, per tree, was demarcated with the variation of wood properties in the pith to bark direction together with linear regression analysis^10,12^. A polynomial model was adjusted by the regression analysis, considering the morphology of the fibers and vessels, specific gravity, tangential and radial shrinkage and microfibril angle (dependent variable) and the position in the pith-bark direction (independent variable). The best equation (fiber length) was chosen according to the significance of the regression analysis and estimated coefficients, coefficient of determination (R²) and the standard error of estimation (Syx).

A graph with the data variability of fiber length in the pith-bark direction was plotted together with the curve of adjusted polynomial model. The comparison of the actual trend of fiber length data along the diameter data aligned to the curve produced by the polynomial model enabled choosing two preliminary inflection points. The fiber length data at radial positions between these points were used to fit a simple linear model. The formed line crossed the polynomial model curve and demarcated the transition zone. The definitive inflection points were defined as those in which the fit presented the highest determination coefficient in simple linear regression.

The data begin to stabilize from the inflection point at the end of the transition zone, and the deviations between the fiber length value in this and subsequent positions was very small, which demarcates the mature wood region. Fits were made for each pith side and presented in a single of diametric variation graph at the end of analysis. The inflection point at the beginning of transition and end zone of juvenile wood was observed as the position of the radius at which the fiber length increased linearly in the juvenile period, the increment decreased or the variation direction changed.

The juvenile, transition and mature wood properties by environment were evaluated by analysis of variance and when significant at 5% significance. The treatment means were compared by the Tukey test at 5%. The proportion of each type of wood in the diameter between upland and floodplain forests was compared by Student’s t-test at 1% level of confidence.

## Data Availability

The datasets generated during and/or analyzed during the current study are available from the corresponding author upon reasonable request.
